# Towards the Millennium of Cybermedicine

**DOI:** 10.2196/jmir.1.suppl1.e2

**Published:** 1999-09-19

**Authors:** Gunther Eysenbach

**Affiliations:** ^1^Department of Clinical Social Medicine, Unit for CybermedicineUniversity of HeidelbergGermany

## Medicine on the Internet

The evolution of the 'information age' in medicine is mirrored in the exponential growth of medical webpages, increasing number of online databases, and expanding services and publications available on the Internet. The handful of computers linked by the predecessor of the Internet in 1969 has grown to more than 5 million websites today, of which at least 100,000 have health-related content. More than 150 million people currently communicate over the Internet. Medical information is among the most retrieved information on the web. Health information providers on the web mostly include private companies offering medical products or medical information, individual patients and health professionals, patient self support groups and professional associations, non-governmental organisations, universities, research institutes and governmental agencies.

## What is Cybermedicine?

The Internet is likely to have a significant impact on the efficiency and quality of future health care, consumer empowerment, public health, medical education and a number of other areas. At the crossroads of medical informatics and public health a new academic field of "cybermedicine" is arising, which can be defined as follows:

"Cybermedicine is the science of applying Internet and global networking technologies to the area of medicine and public health, of studying the impact and implications of the Internet and of evaluating opportunities and the challenges in health care" [1].

The differences between cybermedicine and telemedicine can be highlighted in the following way: Cybermedicine deals with global exchange of open, non-clinical information, mostly from patient-to-patient, sometimes from patient-to-physician and from physician-to-physician, while telemedicine mainly deals with exchange of clinical data in a closed setting. Telemedicine for the most part is applied to diagnostic and curative medicine, while cybermedicine is applied to preventive medicine and public health. Despite these differences, cybermedicine and telemedicine are related disciplines and there are overlapping areas, as increasingly telemedical applications will use the Internet as a platform to deliver and exchange clinical data, as soon as security concerns are resolved.

## The role of consumers

Today, a large number of patients and consumers already use the Internet to retrieve health related information, to interact with health providers and even to order pharmaceutical products. Physicians mainly use the web to access databases such as Medline or to read electronic publications, but in many parts of the (developed) world they clearly lag behind other professions in their use of modern information technology — instead, consumers have taken the lead in adopting the new media for retrieving and exchanging health information. Informed and Internet-savvy patients will play a crucial role in being a major driving force for clinicians to 'go online' and for evidence based medicine: Consumers accessing online information will inevitably increase the pressure on care givers to use timely evidence, and will encourage them to acquaint themselves with information technology to deliver high quality health services. For the first time in the history of medicine, consumers have equal access to the knowledgebases of medicine — and they are making heavy use of this: It has been noted that "the number of Medline searches performed by directly accessing the database at the National Library of Medicine increased from 7 million in 1996 to 120 million in 1997, when free public access was opened; the new searches are attributed primarily to non-physicians" [2]. Thus, the Internet will act as a catalyst for evidence based medicine in two ways: First, it enables health professionals to access timely evidence. Second, it enables consumers to draw from the very same knowledgebase, leading to increased pressure on health professionals to actually *use* the evidence.

## Quality of medical information

The often poor quality of information on the Internet is a potential limiting factor for the usefulness of the Internet — for both consumers and health professionals. Studies assessing the quality of health related websites, newsgroups or evaluating interactive venues using the method of posing as a fictitious patient have demonstrated that important aspects of quality like reliability, accessibility and completeness of information and advice on the Internet are extremely variable. While this problem is also known to happen in traditional media like magazines, newspapers, and television, the Internet creates additional problems, such as the originators of messages and their credibility are difficult to assess by readers. Solutions for these concerns — such as the widespread use of evaluative metainformation — have been proposed [3] and will, once adopted, help to make the web a more useful tool for patient education. Furthermore, the Internet will open new ways for professional medical education.

## Future perspectives

The Internet will not be the same in the next millennium. Important developments are likely to improve not only the speed (Next Generation Internet) but also the quality of information and retrieval possibilities (by linking human-readable with machine-understandable information). The web could evolve into a global medical knowledgebase, where diverse medical Internet applications and resources are interconnected and integrated beyond manual "linking", revolutionising once again knowledge discovery and dissemination in medicine. Moreover, the Internet will (and partly has already begun to) revolutionize science itself by opening new ways of scholarly communication and electronic publishing.

## The need for sound research

The Internet and related new communication technologies enable health professionals to reach large populations with interactive applications, which in turn opens enormous opportunities and challenges. The need for research (and funding) is clear. 'Cybermedicine' research should go beyond mere development and provision of technical solutions; it should also address social and human factors, and evaluate the impact of the Internet on society and health care, and public health. As researchers, we have the responsibility not to follow blindly the general Internet hype but to help physicians and consumers to maximize the use of the Internet by carefully evaluating our interventions and revealing determinants that influence effectiveness and efficiency of Internet communications in health care. Many of these evaluation methods are yet to be developed.

The results of MEDNET'99 and the publication of the presentations in this abstract book (as well as publication of the best papers of this congress in a forthcoming special issue of the Journal of Medical Internet Research) will hopefully provide a primer and a stimulus for thought and research about the utility and impact of the Internet on health care in the next millennium.

## References

[1] Eysenbach G, Sa E R, Diepgen T L (1999). Shopping the Internet today and tomorrow - Towards the Millennium of Cybermedicine. BMJ.

[2] Sieving P C (1999). Factors driving the increase in medical information on the web--one American perspective. J Med Internet Res.

[3] Eysenbach G, Diepgen T L (1998). Towards quality management of medical information on the internet: evaluation, labelling, and filtering of information. BMJ.

